# Highly efficient synthesis of pyrimidine-5-carbonitrile derivatives over a robust biowaste bone char-Bronsted solid acid catalyst

**DOI:** 10.1038/s41598-024-82040-3

**Published:** 2024-12-06

**Authors:** Zahra Siahpour, Maryam Hajjami

**Affiliations:** Department of Organic Chemistry, Faculty of Chemistry and Petroleum Sciences, Bu-Ali Sina University, Hamedan, 6517838683 Iran

**Keywords:** Bone char, Pyrimidine-5-carbonitrile, Nano catalyst, Green catalyst, Hydroxy apatite, Catalysis, Organic chemistry

## Abstract

In this study, the conversion of bones (waste of food industry) into bone char is described. The presence of calcium phosphate and graphitic carbon gives bone char unique properties, with different possible uses. The catalytic behavior of bone char modified with chlorosulfonic acid is tested as reusable and eco-friendly solid acid biocatalyst in synthesis of pyrimidine-5-carbonitrile derivatives. The catalyst properties were characterized by different techniques such as FT-IR, BET, SEM, TEM, TGA, EDS and XRD. The bone char catalyst can be reused several times without reducing catalyst efficiency. In addition to the various reported benefits, the first use of bone char as a catalyst support for organic synthesis is an important novelty of this study.

**Supplementary Information:**

The online version contains supplementary material available at 10.1038/s41598-024-82040-3.

## Introduction

Materials with ordered porous properties at the nanoscale have important uses in different fields such as optics, drug delivery systems, catalysis, coatings, cosmetics, diagnostics, bio separation, nanotechnology, and air separation. Most nano-porous materials fall into three broad categories: micro, meso, and macro^1^. Mesoporous materials are of vast attention in the fields of science and technology due to their many advantages, including large surface area and high pore volume, greater accessibility, and the ability to adapt and interact with different chemical functions on their surface^2,3^. Porous carbon materials are known to be very effective adsorbents. In particular, those obtained from biological waste have been widely studied in recent years because of their outstanding effectiveness in removing organic pollutants and heavy metals^4–8^. In this context, low-cost carbon materials derived from natural-based based have particular interest.

Biochar is a heterogeneous carbon material consisting of some functional groups of many levels, which is produced by the thermal conversion of various types of waste. Biochar is usually a carbon-rich product obtained when different biomass such as wood, manure, leaves, sludge, municipal sewage, etc. is heated in a closed system with slight or no oxygen^9^. also, Animal bone char is another produce of carbon-containing material that can be produced under similar controlled thermal conditions to produce a phosphorus-rich product^10^. Bone char is a solid material that is produced from the pyrolysis of animal bones and is usually used as a decolorizing adsorbent in refined sugar. During this process, commonly the bone is heated in an inert or oxidized atmosphere at a temperature between 400 and 600 ^0^C, for control of the quality and efficiency of the product^11^.

Bone char is usually composed of two materials carbon and hydroxyapatite (HAP) [Ca_10_ (PO_4_)_6_ (OH)_2_]^12^. It is constituted by calcium hydroxyapatite (70–76%), carbon (9–11%), and calcium carbonate (7–9%) and has a surface area of approximately 100 m^2^ g^− 1^ with a structure composed exclusively of mesoporous^13–15^. Further pore diameter detection shows that the pore sizes of bone char range from 1.7 to 75 nm, including major mesopores (2 to 50 nm) and a small number of pores with a diameter of 50 nm (macropores)^16^. Bone char has attracted attention due to its cheapness, ease of production, and biocompatibility^17^. Various reactions such as degradation, condensation, and hydrogen removal are possible and cause “accumulation” of these materials on the surface of hydroxyapatite crystals^18^. Among the materials utilized as adsorbents, usually bone char has been widely used to remove various pollutants in drinking water^19,20^.

Heterocyclic compounds are of biological attentiveness due to their potential chemical and physical properties^21^. Among these, pyrimidine derivatives have attracted considerable attention for their pharmacological properties such as bactericidal, fungicidal^22^, analgesic^23^, anti-inflammatory^24^, anti-cancer^25^, antioxidant^26^, and anti-HIV^27^. The synthetic diversification of pyrimidine allows the production of a variety of derivatives, including analogs resulting from aryl ring substitution, pyrimidine nitrogen derivatization, and carbon substitution at the 2, 4, 5, and 6 positions^27^. Although some routes of pyrimidine synthesis have been known for a long ago, the development of other more economical routes is of substantial importance^28,–30^.

In continuation of our investigation on biowaste-based catalysis systems^31–35^, In this work, we develop an efficient method for one-pot synthesis of pyrimidine-5-carbonitrile derivatives by condensation of three components including aromatic aldehydes, malononitrile, and thiourea or urea in the presence of bone char-nPrN-SO_3_H as a recyclable nanocatalyst in solvent-free conditions at 80 °C. The results obtained from the study of the synthesis of pyrimidine-5-carbonitrile showed excellent yield and reduced reaction time to less than a few minutes.

## Materials and methods

### Materials and methods

The chemicals used in this research were acquired from Sigma-Aldrich and employed without undergoing additional purification. The XRD pattern was carried out using a PANalytical. Data were recorded in the diffractometer with CuKα radiations in diverse angle range 2 ≤ °2θ ≤ 90, operating at 40kv and 30 mA. Instruments used in Brunauer-Emmett-Teller have this character with Belsorp company, Japan. In order to observe changes in the functional groups, the FTIR spectra of the materials were collected using a KBr pellet and a Nicolet MAGNA-IR 550 spectrometer (Madison, WI, USA). The transmission electron microscopy (TEM) technique was employed with a TEM Philips EM 208 S instrument, operating at an accelerating voltage of 100 kV, to analyze the morphology and particle size. X-ray energy dispersive spectroscopy (EDX) was employed to determine the elemental composition of a sample. Thermogravimetric analyses (TGA) of the samples were recorded using a Shimadzu PL-STA 1500 device in the temperature range 30–800 °C used to study the thermal behavior of materials. To monitor the temperature of a synthesized product the melting points were measured using the electrothermal IA9200 apparatus. The reaction progress and evaluation of substrate purity were monitored using thin-layer chromatography (TLC) on silica gel SILG/UV 254 and 365 plates.

### Preparation BC

first bovine bones were crushed into 1–3 cm pieces, then washed three times in hot deionized water for 4 h to remove fat and protein residues, the achieve bones pieces were dried overnight at 110 °C. Bone pyrolysis was performed in an air (oxygen)-free furnace at 450 °C for 4.5 h with a heating rate of 10 °C/min to produce bone char. The bone char was allowed to cool to room temperature, then crushed and sieved to obtain particles with a diameter of 0.2 mm. Finally, the obtained sample is stored in a sealed container.

### Functionalization of bone char surfaces with 3-aminopropyltriethoxysilane

Firstly 6 gr, 3-aminopropyltriethoxysilane was added in 50 ml of the ethanol/water (9/1 Stir in a 100 ml flask at room temperature (25 ± 2 °C)) with a magnetic stirrer for approximate 30 min. Then 3 g of bone char obtained from the previous step was added to the solution and stirred at reflux condition for 24 h. Finally, the functionalization of bone char was washed for several times with water (H_2_O) and ethanol (EtOH) and dried at room temperature.

### Bone char-nPrN-SO3H

Accurate 0.5 g of functionalization of bone char powder was dispersed in sonicated in 20 mL of dichloromethane) CH_2_Cl_2_) for 20 min. Then 2 ml of chlorosulfonic acid (ClSO_3_H) was added dropwise and stirred in an ice bath for 3 h. Finally, the synthesized nano catalyst was washed for several time with water (H_2_O) and ethanol (EtOH) and dried at room temperature. Also, to determine the amount of acid in catalyst according to the literature,13 the 0.1 g of catalyst was added to an aqueous NaCl solution (1 mol/L, 10 mL) with an initial pH 7.1. The mixture was stirred for 30 min until the pH of solution decreased to 3.1 that indicating an ion exchange between sulfamic acid protons and sodium ions and this is equal to a loading of 0.079 mmol.g^− 1^ of sulfamic acid group.

### General procedure for the synthesis of pyrimidine-5-carbonitrile derivatives

A mixture of aldehyde derivatives (1 mol), urea/thiourea (1.8 mol), malononitrile (1.2 mol) and bone char-nPrN-SO3H (0.4 mol%) were placed in a round-bottom flask in solvent-free conditions at 80 °C, then mixture was stirred for the appropriate time. After the reaction was completed (by TLC thin layer chromatography), distilled water was added to the reaction mixture and cooled to room temperature. The resulting precipitate was separated by filtration and washed with hot ethanol (3 × 2 ml). The precipitate was then recrystallized from ethanol giving the pure product.

### Selected NMR data

6-Amino-4-(4-boromophenyl)-5-cyano-2-hydroxypyrimidine: ^1^H NMR (250 MHz, DMSO-d6) 8.62 (s, 2 H), 8.03 (d, J = 8.5 Hz, 2 H), 7.90 (s, 1H), 7.70 (d, J = 8.6 Hz, 2 H).^13^C NMR (63 MHz, DMSO-d6) δ 157.2, 139.2, 135.8, 131.4, 130.5, 128.8, 113.8, 112.7, 87.5. IR (KBr): 3422 (cm^− 1^) (broad, OH), 3091 (cm^− 1^) (NH_2_), 2227 (cm^− 1^) and 1639 (cm^− 1^) (CN).

6-Amino-4-(2,4-dichlorophenyl)-5-cyano-2-hydroxypyrimidine: ^1^H NMR (250 MHz, DMSO-d6) δ 8.51 (s, 2 H), 8.19–7.54 (m, 4 H). ^13^C NMR (63 MHz, DMSO-d6) δ 182.3, 160.7, 134.8, 134.4, 133.1, 132.6, 130.8, 128.8, 114.5, 113.4, 82.8. IR (KBr): 3422 (cm^− 1^) (broad, OH), 3102 (cm^− 1^) (NH_2_), 2229 (cm^− 1^) and 1639 (cm^− 1^) (CN).

6-Amino-5-Cyano-4-(4-boromo)-Phenyl-2-MercaptoPyrimidine: ^1^H NMR (250 MHz, DMSO-d6) δ 8.50 (s, 2 H), 7.86 (d, J = 7.4 Hz, 4 H), 2.71 (s, 1H). ^13^C NMR (63 MHz, DMSO-d6) δ 178.5, 162., 160.7, 133.1, 132.6, 130.8, 128.8, 114.5, 84.5, IR (KBr): 3410 (cm^− 1^) (NH_2_), 3032 (cm^− 1^) (C-H), 2227 (cm^− 1^) and 1639(cm^− 1^) (CN).

## Result and discussion

### Catalyst characterization

To gain insight into the properties of bone char-nPrN-SO_3_H synthesized as Bronsted acid from cattle bone, FTIR, SEM, TEM, EDX, and XRD analysis was performed and discussed as follows.

#### SEM analysis

**Fig. 1:**
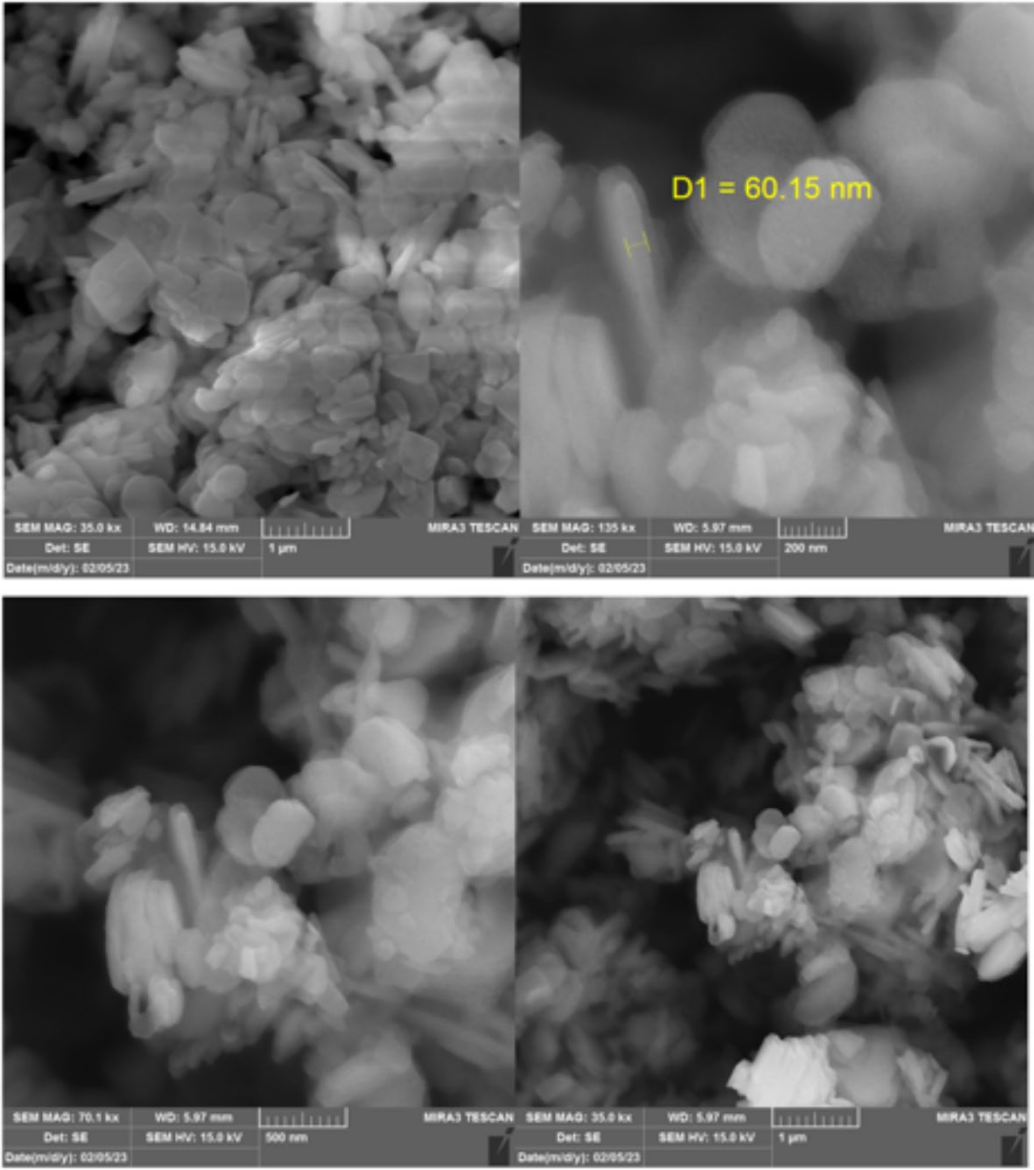
SEM image of modified bone char as Bronsted acid catalysis.

**Fig. 2:**
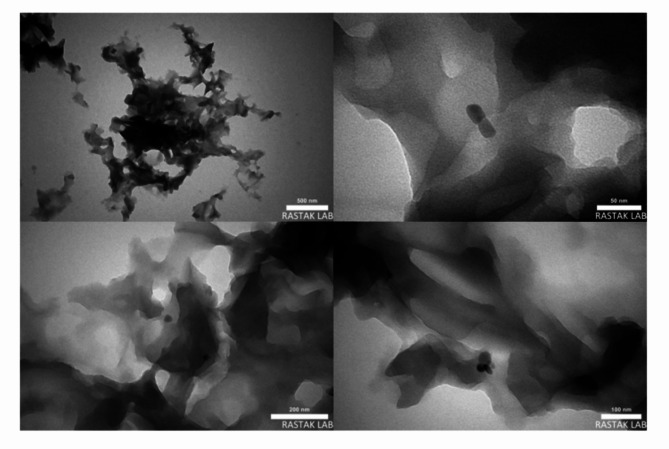
TEM image of modified bone char as Bronsted acid catalyst.

Scanning electron microscopy (SEM) effectively examines the surface structures and morphological characteristics of synthesized catalysts. The SEM image of modification bone char shown in Fig. 1. The modified bone char, used as a Brønsted acid catalyst, was characterized using SEM to assess its morphology and typographical structure. The SEM images of the bone char -nPrN-SO_3_H catalyst reveal rough and uneven surface structure with irregular flaky particles of varying sizes that are stacked and agglomerated due to their small dimensions. The SEM images in surface display small amount of apparent porosity that confirming their nanoscale size. Also, the transmission electron microscopy (TEM) images show rice and needle-like crystal shapes^36^. Rice-shaped particles have small diameter and length, while needle-shaped particles have small diameters, longer lengths, and a uniform morphology. The TEM images of bone char -nPrN-SO_3_H catalyst is shown in Fig. 2.

#### XRD analysis

**Fig. 3:**
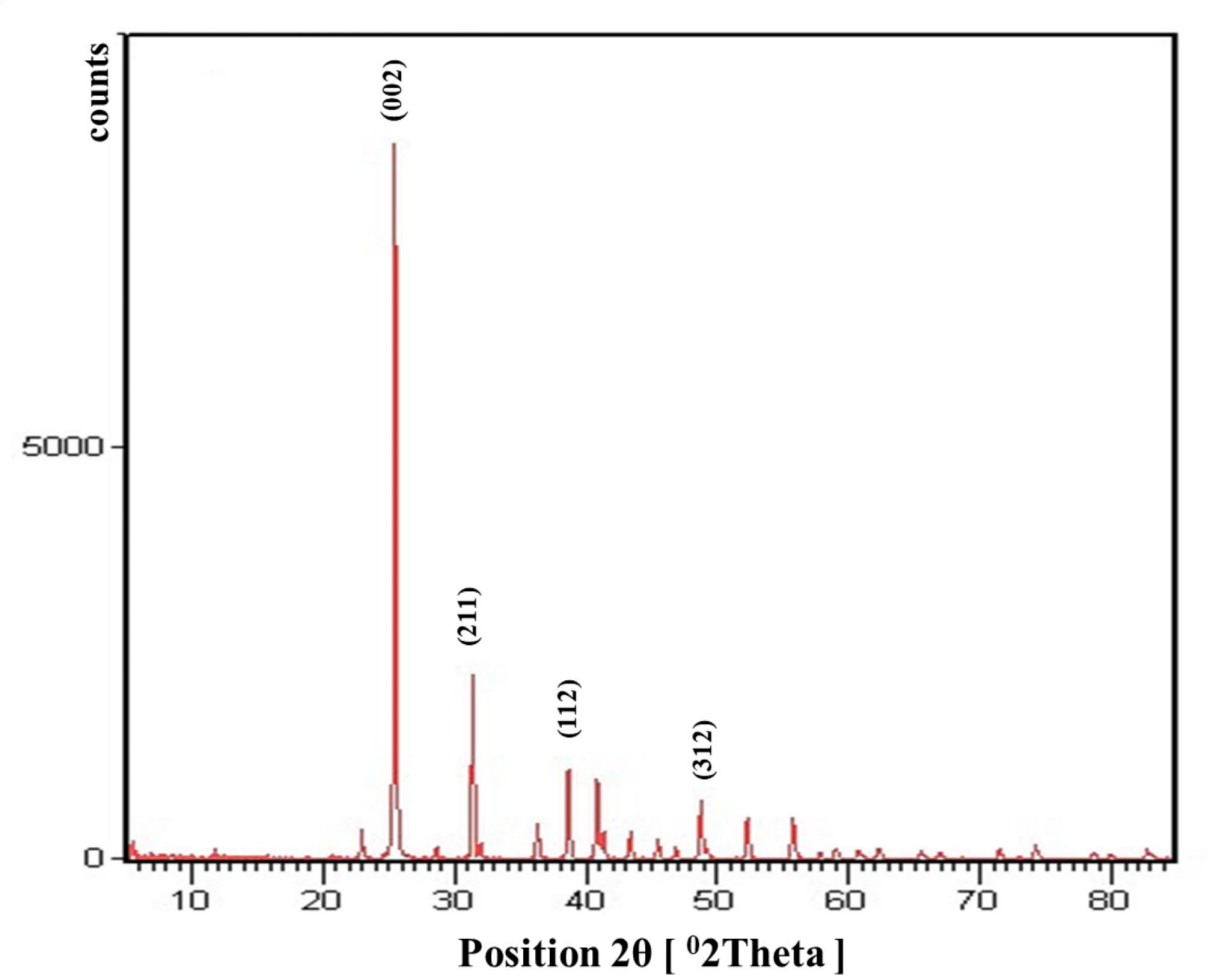
XRD pattern of bone char -nPrN-SO_3_H catalyst.

The XRD study was performed on modified bone char as shown in Fig. 3. The pattern obtained corresponds to the standard pattern of hydroxyapatite crystals. Diffraction peaks were observed at 2θ (degree) of 25.4°, 28.6°, 31.3°, 32.0°, 43.3° and 48.7° were appeared in good match with the crystal planes of composition hydroxyapatite (002), (211), (112) and (312), respectively. Sharper peaks observed in the XRD pattern indicate better crystallinity. Also, the peak observed at 25.4º is relates to the c-axis of the hydroxyapatite structure^37^. The diffraction of plane 002 and 211 results in the highest intensity 25.4º and 31.3º than all other peaks in obtained pattern^33^. In addition, the dihydroxylation reaction of hydroxyapatite can also cause the peak shift of XRD patterns^38^.

#### BET analysis (surface area and porosity analysis)

BET is the most commonly used method to measure the surface area of a porous compound using N_2_ gas as an adsorbate on the material surface. The specific surface properties of catalyst obtained at a pyrolysis temperature of 450 °C were determined using N_2_ adsorption at 77.35 K and a relative pressure range (P/Po) from 0.0 to 1. According to Brunauer-Emmett-Teller (BET), the specific surface area of the modified bone char sample obtained 61.15 m^2^ /g with a pore volumes 0.69 cm^3^/g, and average pore diameter 45 nm.

**Fig. 4:**
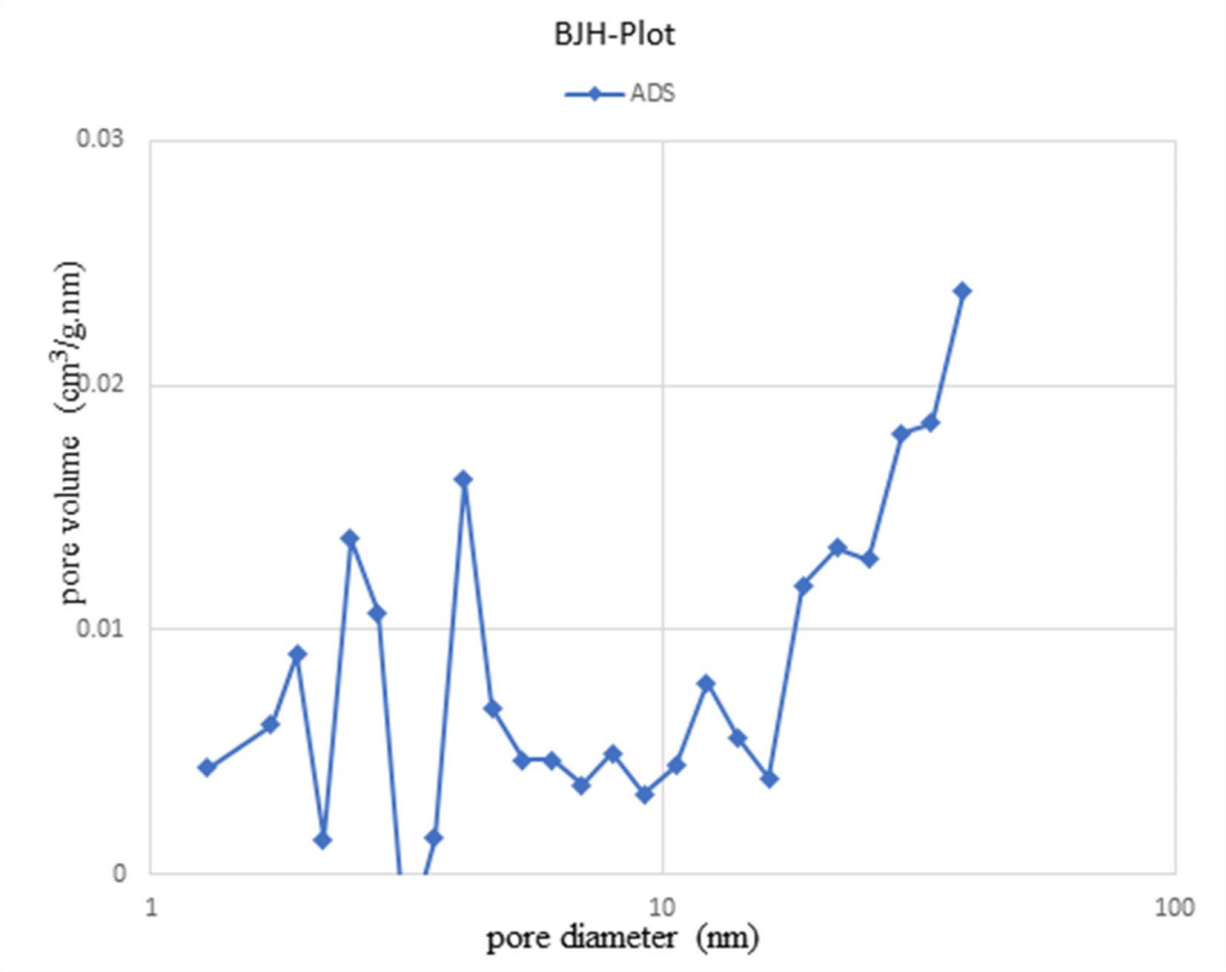
BJH adsorption dV/dr Pore volume distribution for bone char-nPrN-SO_3_H obtained from pyrolyzed of Cattle bones at 450 °C temperatures with residence time 4.5 h.

The BJH (Barrett, Joyner, Halenda) method was used to calculating the pore size distribution of the adsorbent. Figure 4 shows the pore size distribution curve of the synthesized bone char sample in this work. The curves show the pore size distribution in the micropore (< 2 nm), mesopore (2–50 nm) and macropore regime (> 50 nm). The analysed sample ranges from 1.2 to 38.52 nm and is mainly distributed in mesoporous mode.

**Fig. 5:**
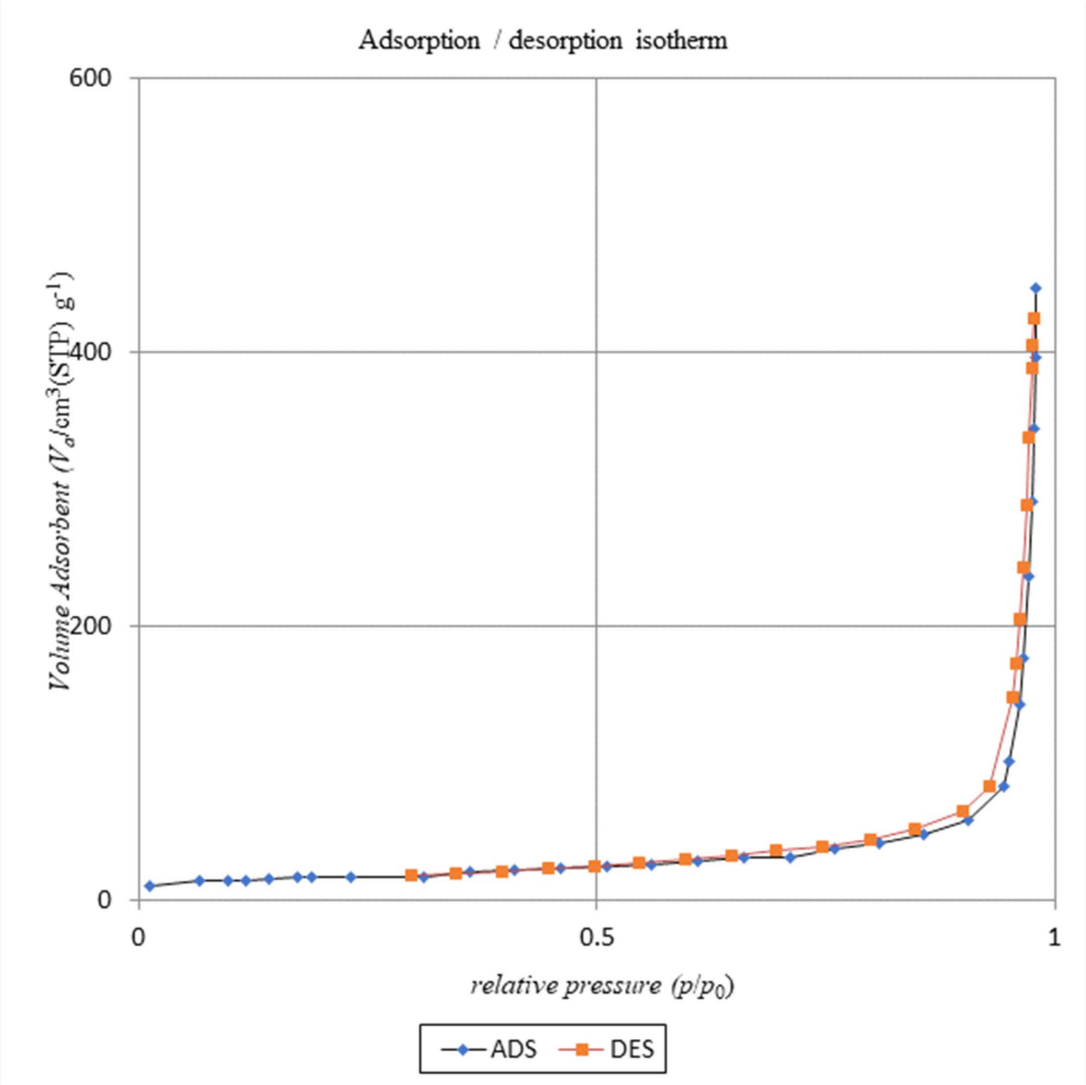
Nitrogen adsorption isotherms for bone char-nPrN-SO_3_H obtained from pyrolyzed of Cattle bones at 450 °C temperatures with residence time 4.5 h.

Nitrogen adsorbed at relatively low pressure indicates that thermal decomposition and operating conditions in pyrolysis process may partially cause the formation of micropores, and this hypothesis can be confirmed by the obtained isotherm illustrated in Fig. 5. Table 1 shows a brief comparison from parameter such as surface area and Pore volume in this work with other results available in the literature^39^.

**Table 1:** Comparison of operating parameters on various types of bones.

Source	Operating parameters	Surface area (m^2^/g)	Pore volume (cm^3^/g)	Ref.
Cattle bones	Temp-600 °C, Rate of heating-10 °C/min, residence time-2 h, pyrolyzed in a rectangular furnace	50.4	0.305	40
Cattle bones	Temp-600 °C, Rate of heating-10 °C/min, residence time-1 h, pyrolyzed in rectangular muffle furnace	57.9	0.293	40
Cattle bones	A commercial BC was utilized in this study and was fixed by calcining cattle bones by the APELSA company, Mexico.	75	0.22	41
Sheep bones	Temp-900 °C, Rate of heating-10 °C/min, residence time-2 h, pyrolyzed in a rectangular furnace	38	0.16	42
Cattle bones	Temp-450 °C, Rate of heating-10 °C/min, residence time-4.5 h, pyrolyzed in a rectangular furnace	61.15	0.69	This work

#### FT-IR spectra

**Fig. 6:**
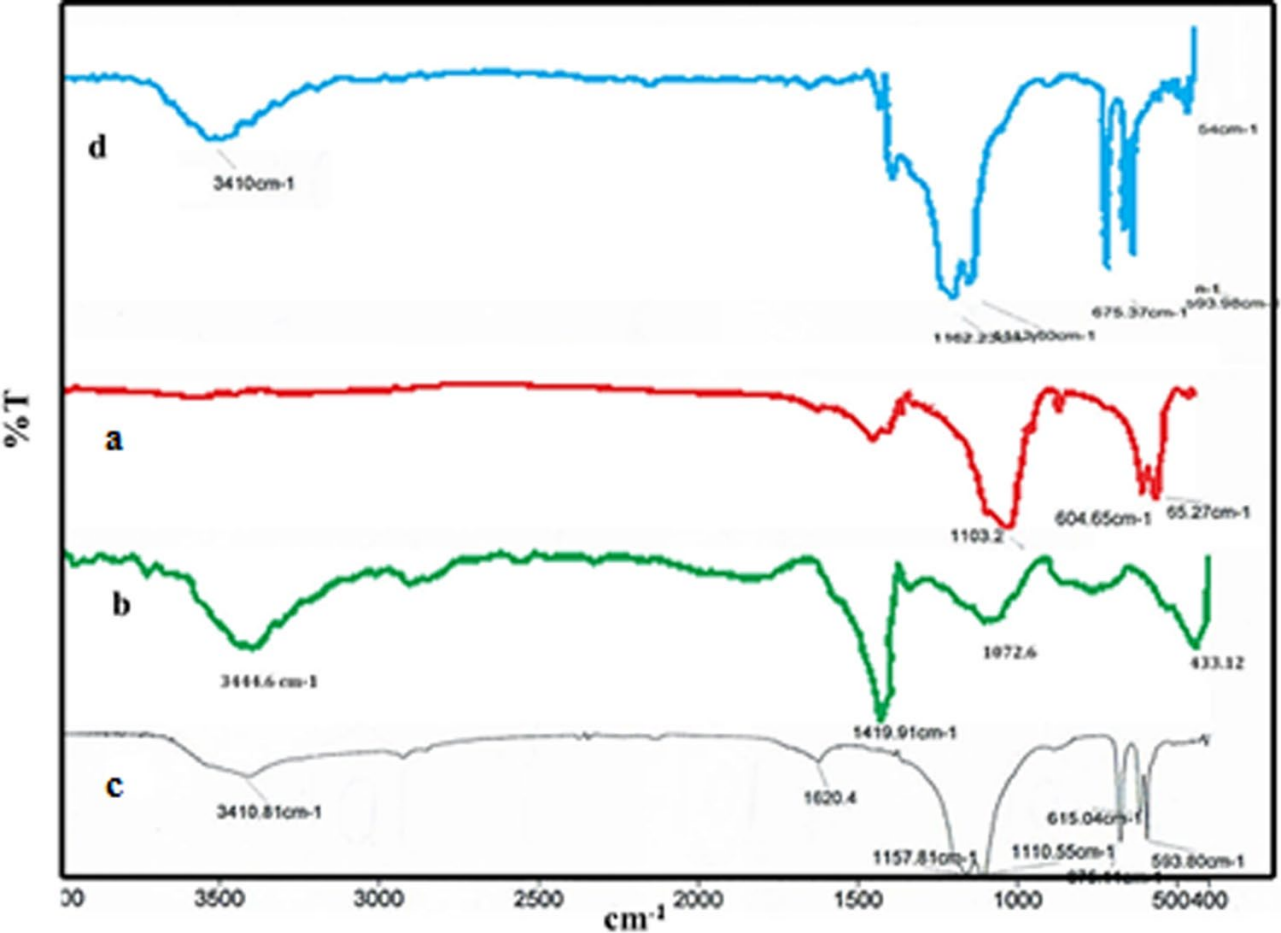
FT-IR spectrum of modified bone char catalyst.

Figure 6 shows the FT-IR spectra of bone char (a), bone char-3-aminopropyl-trietoxysilan (b), bone char-nPrN-SO_3_H (c) and recovered bone char-nPrN-SO_3_H (d). In Spectrum (a) the peak observed at 1470 cm^− 1^correspond to CO_3_ ^− 2^ group, intensive PO_4_ ^− 3^ in at 1031 cm^− 1^ and weak C-H in at 870 cm^− 1^. The observed peaks at 1031 and 961 cm^− 1^ are attributed to the vibrational stretching of the PO_4_ ^− 3^ group. The peaks observed at 604, 565 and 472 cm ^− 1^ correspond to the bending vibrations of PO_4_ ^− 3^.

**Fig. 7:**
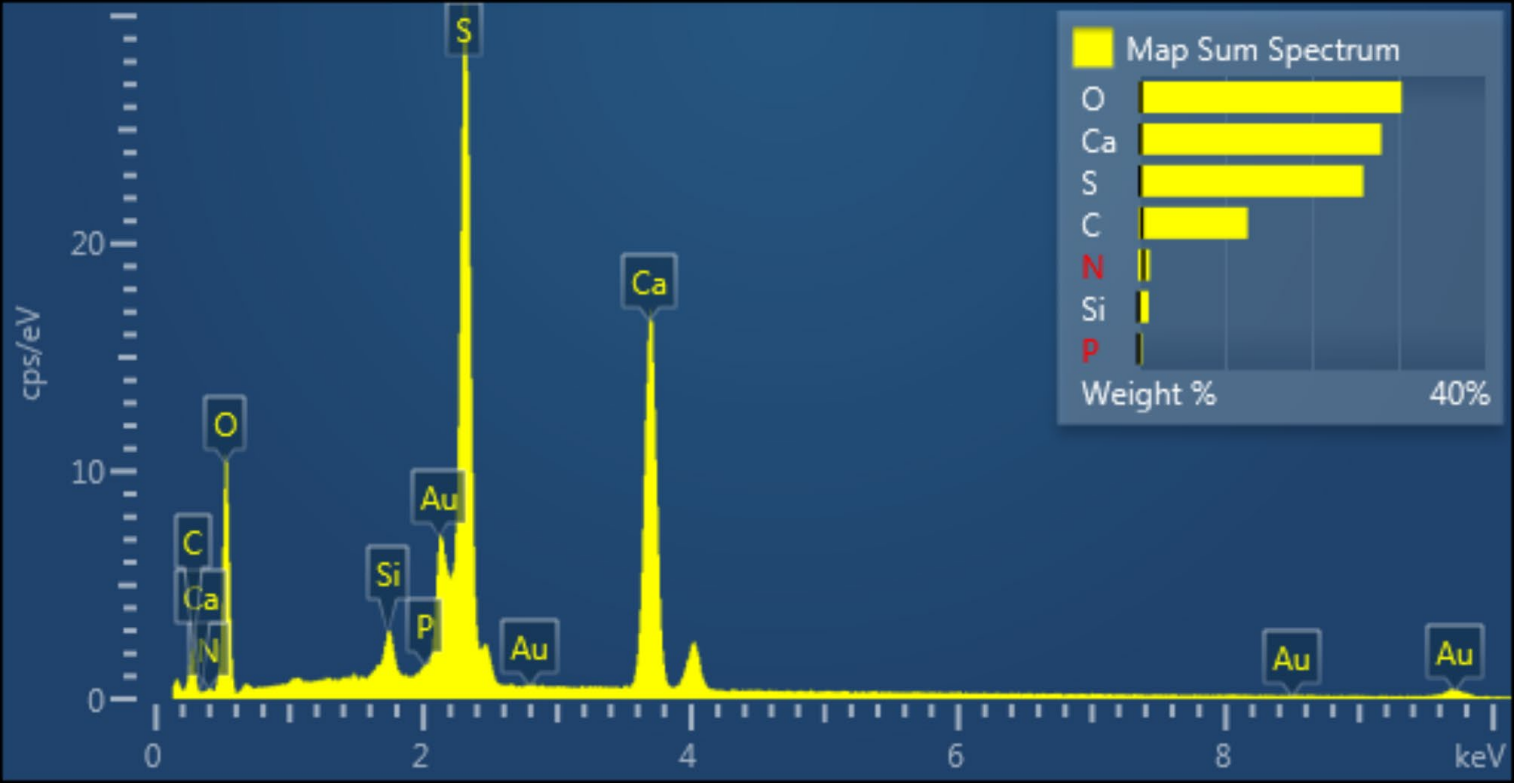
EDS diagram of Modified bone char catalysis.

After functionalization of Bone char with 3-aminopropyl-trietoxysilan (Fig. 7. spectrum (b)) vibration bands of stretch and bend aliphatic CH_2_ groups, in 1420 cm^− 1^ and 2988 cm^− 1^, were observed. Also, peaks were appeared as characteristic Si-O at 1074 cm^− 1^and as weak O-H at 3411 cm^− 1^. The peak at 803 cm^− 1^ may be due to the symmetric starching vibration of Si-O.

The introduction of sulfonic group into the bone char-nPrN-SO_3_H is evident from the intense peak, in the Fig. 7 spectrum (c), at 1108 cm^− 1^ and 1161 cm^− 1^due to the stretching of S = O double bound as well as the broad band at about 3400 cm^− 1^ assigned to the stretching of the acidic hydroxide (O-H) group.

The FT-IR spectrum of the recovered bone char-nPrN-SO_3_H was compared with the fresh one shown in the spectrum in Fig. 7(d). The FT-IR spectra of fresh and recycled catalyst are in good agreement. These results indicate the stability of bone char-nPrN-SO_3_H under reaction conditions.

#### EDS analysis

**Fig. 8:**
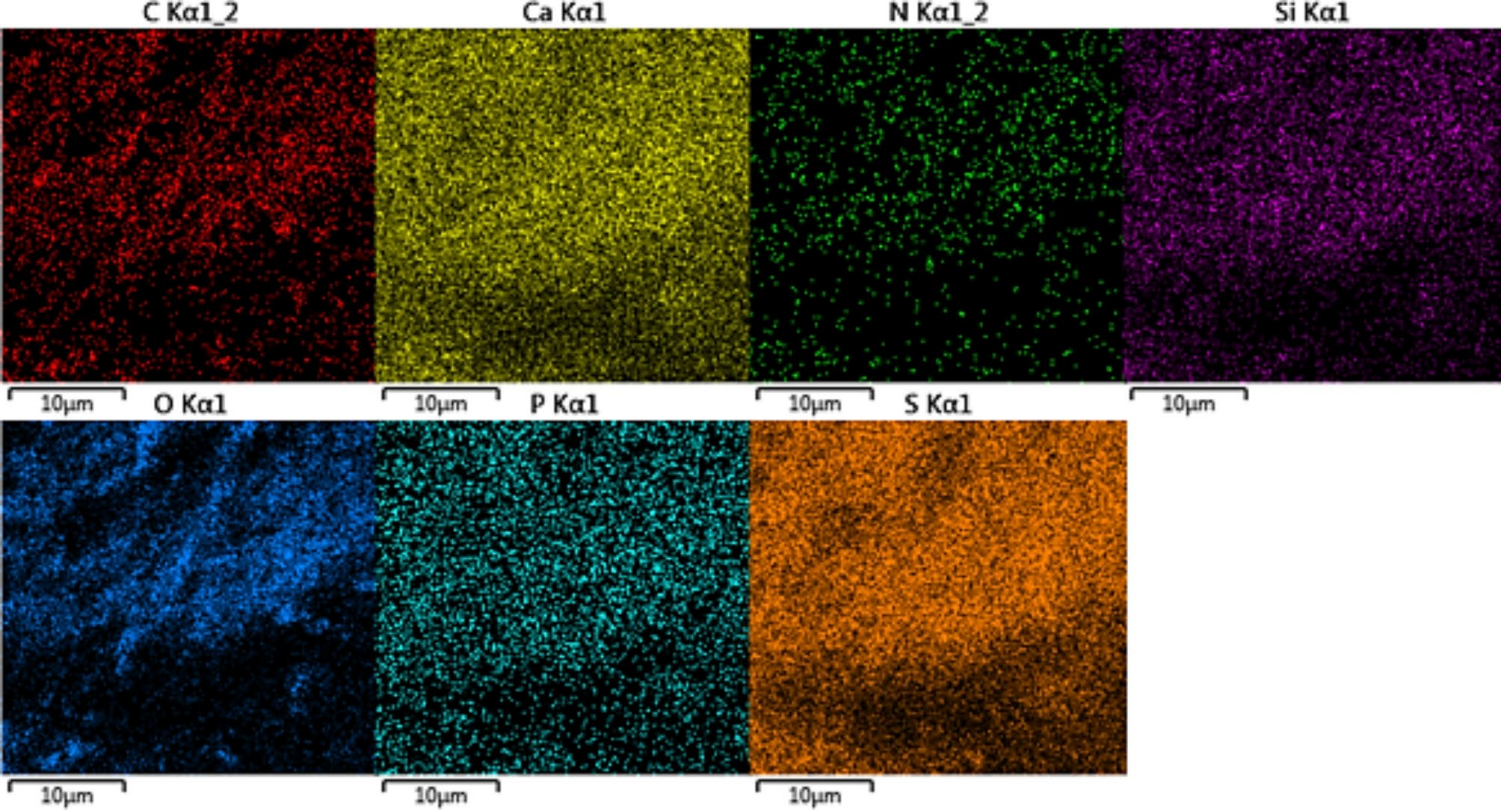
Elemental mapping of carbon, calcium, nitrogen, silica, oxygen, phosphorus and sulfur for modified bone char.

**Fig. 9:**
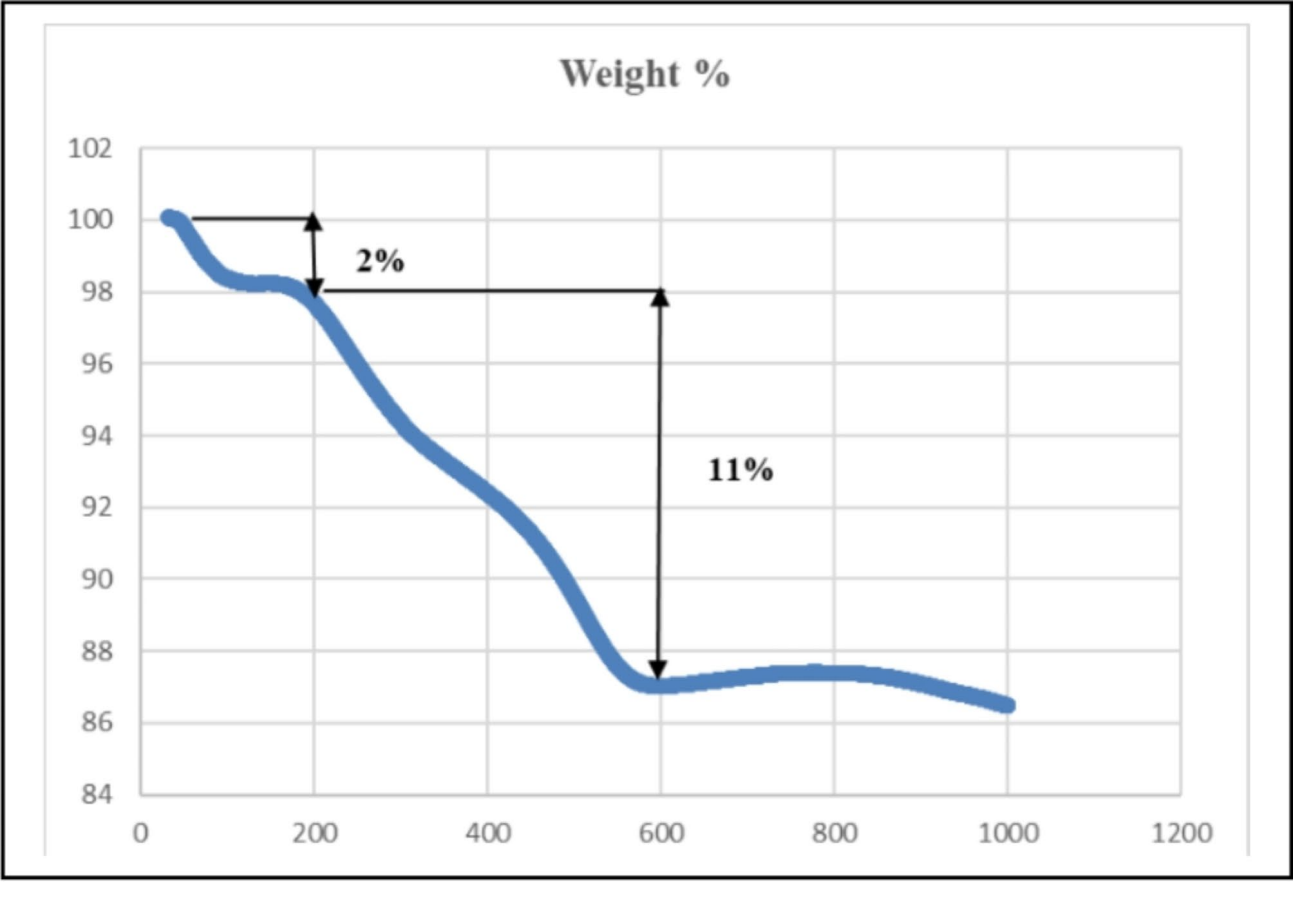
The TGA diagram of bone char-nPrN-SO_3_H as Bronsted acid catalysis.

EDS analysis was performed to confirm the elemental content of bone char-nPrN-SO_3_H obtained at 450 °C (Fig. 7). As seen, the EDS results of modified bone char indicate elements content include calcium (Ca), phosphorus (P), oxygen (O), carbon (C), nitrogen (N), sulfur (S) and silica (Si). Also, Fig. 8 was shown WDX analysis of bone char-nPrN-SO_3_H as Bronsted acid catalysis. According to Fig. 9, the uniform distribution of oxygen, nitrogen, carbon, silica, calcium, sulfur, and phosphorus was clearly observed in the WDX analysis of Modified Bone Char. By seeing Calcium (Ca), phosphorus (P) and oxygen in SEM-EDS of modified bone char verified the presence of hydroxyapatite composition.

#### TGA analysis

The TGA diagram of bone char-nPrN-SO_3_H is shown in Fig. 9. The low weight loss of about 2% at temperatures below 100 °C can be attributed to the evaporation of solvents on the surface of the bone char. Considering that the bone char support is made of bone pyrolysis at a temperature of 450 °C with residence time of 4.5 h, it can be said that the observed weight loss at the temperature 200–600 °C usually due the organic contents (about 11%) were decomposed. This result is strong evidence of the organic layer on the surface of bone char. The TGA curve shows minimal weight loss between 600 and 1000 °C, indicating the stability of the bone char support at high temperatures.

### Catalytic activity in synthesis of pyrimidine-5-carbonitrile derivatives

**Fig. 10:**
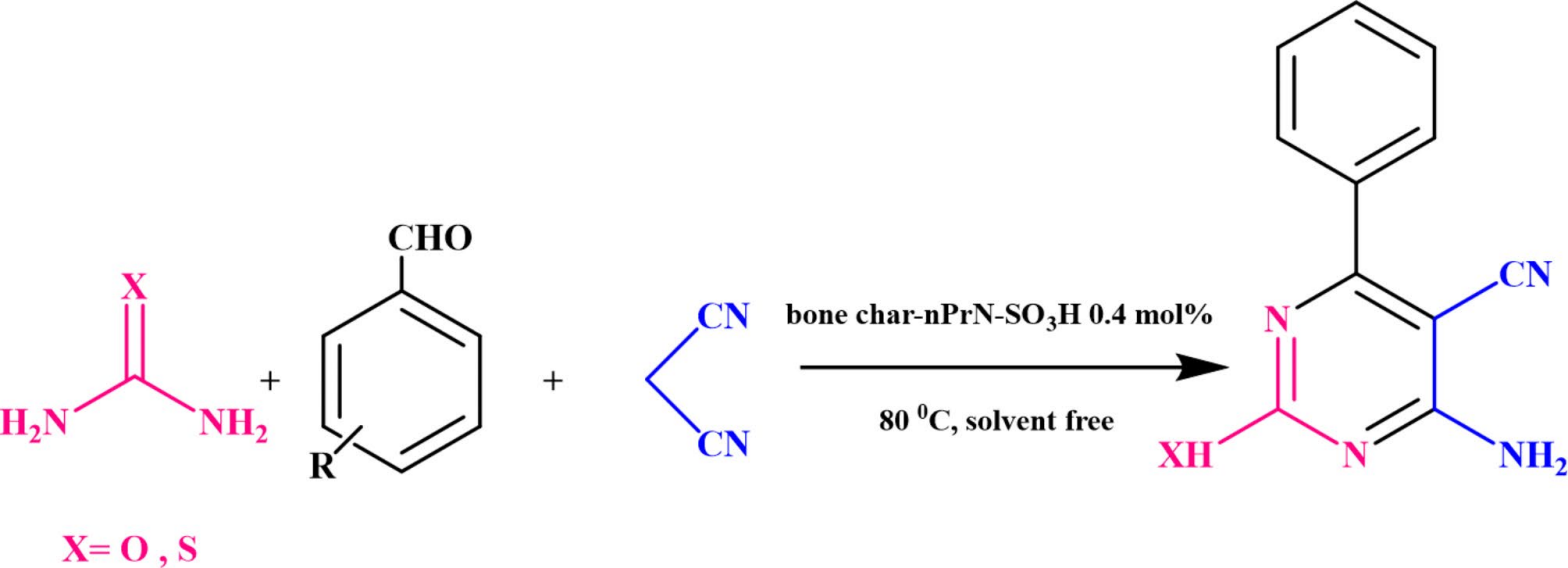
Synthesis of pyrimidine-5-carbonitrile derivatives in the presence of bone char-nPrN-SO_3_H as catalyst in in solvent-free conditions at 80 °C.

First, the modified bone char catalytic activity was investigated in the synthesis of pyrimidine-5-carbonitrile derivatives (Fig. 10). Then, the reaction parameters such as temperature, the catalyst amount and solvent were optimized in the reaction of 4-**c**hlorobenzaldehyde (1 mol), malononitrile (1.2 mol) and urea (1.8 mol) as a model reaction. The results are summarized in Table 2. It was concluded that solvent-free condition at 80 °C in the presence 0.4 mol% bone char-nPrN-SO_3_H as the catalyst is the optimized condition for this three-component reaction.

**Fig. 11:**
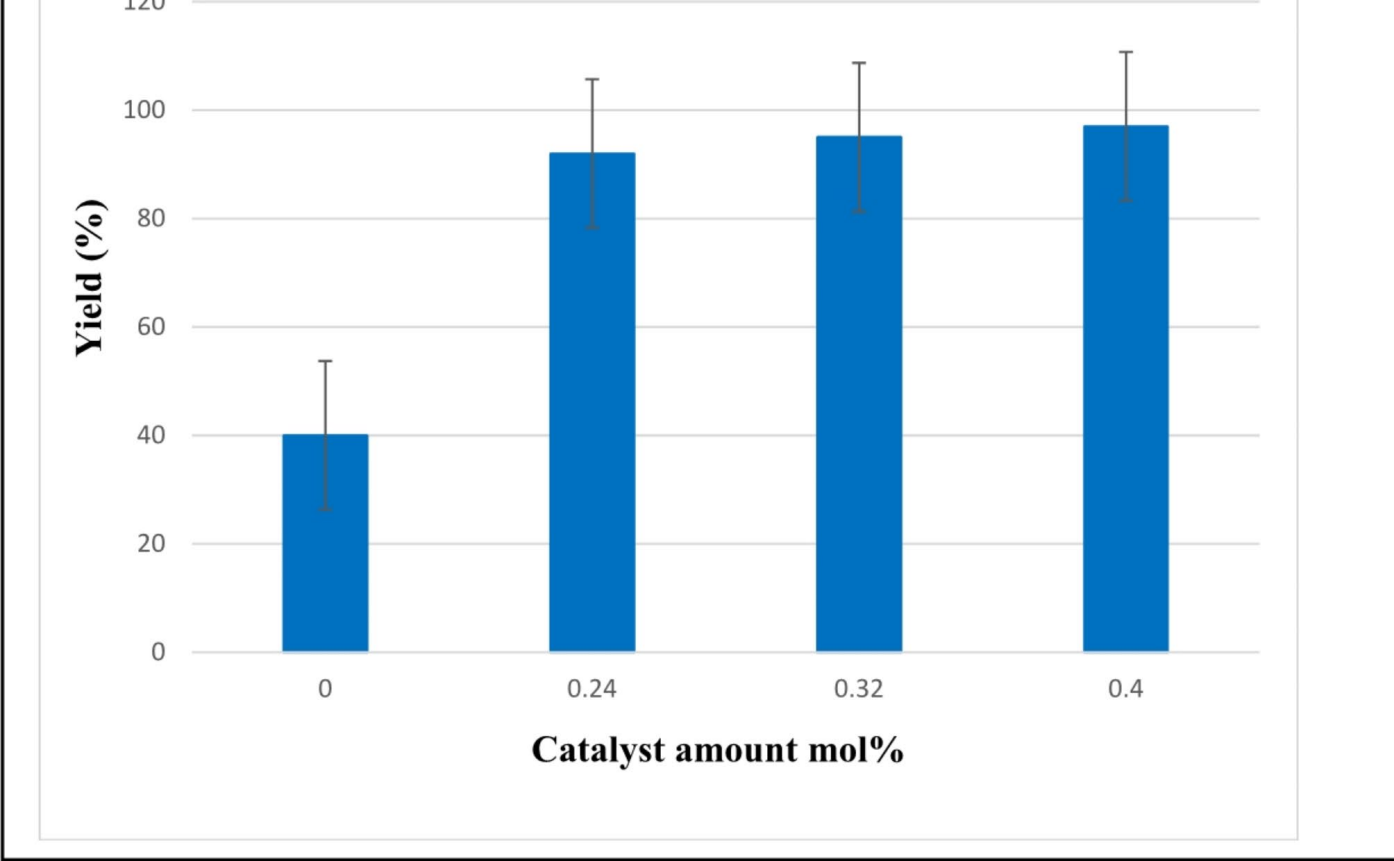
Catalytic performances synthesis of pyrimidine-5-carbonitrile catalyzed by bone char-nPrN-SO_3_H at different amounts of catalyst. Error bars indicate the range of data based on repeat experiments.

In this study, we described a simple, eco-friendly and economical strategy for the synthesis of pyrimidine-5-carbonitrile. After optimizing the reaction conditions, a highly efficient one-pot synthesis of three-component pyrimidine-5-carbonitrile derivatives were also established using the same method. (Table 3). The reaction of various aromatic aldehydes with malononitrile and urea/thiourea was carried out according to the optimized general experimental procedure. As shown in Table 3, the one-pot three-component reaction are performed efficiently in the presence of presence modified bone char-nPrN-SO_3_H as Bronsted acid catalysis with various arylaldehydes containing electron-withdrawing and electron-donating groups, such as Cl, Br, Me, NO_3_, etc., and the yields of most products is over 90%. The great advantage of this system, which distinguishes it from other reported methods, is the very short reaction time. The study of turnover number (TON) and turnover frequency (TOF) in the catalytic system is a very significant area of research which evaluate the efficiency of the catalyst. They refer to the molar number of catalytic sites that converts reactants to products. In this work, TON and TOF were measured for all products and is given in the Table 3. The graphical Error bars is displayed in Fig. 11 to indicate the error bars for different amounts of catalyst.

**Table 2:** Optimization for the synthesis of pyrimidine-5-carbonitrile with 4-chlorobenzaldehyde, urea, malononitrile and bone char-nPrN-SO_3_H as catalyst.

Entry	Catalyst (mol%)	Solvent	Temperature (°C)	Time (min)	Yield%
1	0.00	-	80	240	40
2	0.24	-	80	65	92
3	0.32	-	80	40	95
4	0.4	-	80	5	97
5	0.4	Water	Reflux	240	75
6	0.4	Acetonitrile	Reflux	240	50
7	0.4	n-hexane	Reflux	240	35
8	0.4	Ethanol	Reflux	240	67
9	0.4	Ethanol: water 50:50	Reflux	240	71
10	0.4	-	30	60	45
11	0.4	-	50	35	67
12	0.4	-	70	15	85

**Fig. 12:**
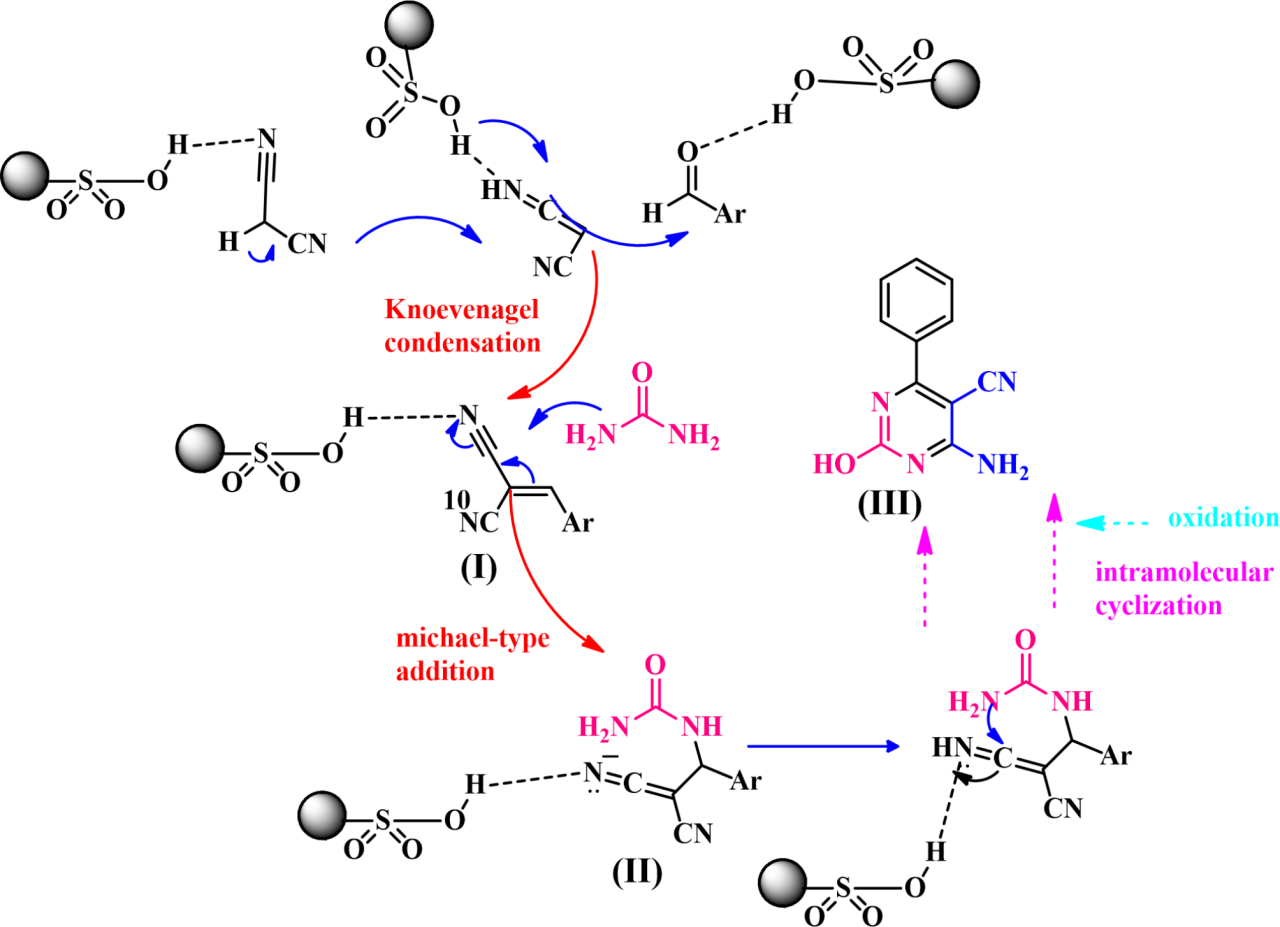
The possible mechanism for the synthesis of pyrimidine-5-carbonitrile in the presence of bone char-nPrN-SO_3_H as catalyst.

The suggested mechanism for the synthesis of pyrimidine-5-carbonitrile in the presence of bone char-nPrN-SO_3_H as catalyst, on the basis of the literature^43^, is proposed in Fig. 12. The condensation of the malononitrile with activated aldehyde by the Knoevenagel reaction give intermediate (I). Then thiourea/urea reacts with (I) by Michael addition to afford the intermediate (II). Finally, cyclodehydration of (II) and tautomerization/aromatization produce pyrimidine-5-carbonitrile (III)^40^.

**Table 3:** Synthesis of pyrimidine-5-carbonitrile derivatives in the presence of bone char-nPrN-SO_3_H as catalyst under solvent-free conditions at 80 °C.

Entry	*R*	x	Time (min)	Yield (%)	TON	TOF min^− 1^
1	H	O	12	91	227	1137
2	4-Cl	O	5	97	242	2921
3	4-NO_2_	O	15	93	232	930
4	2,4-Cl_2_	O	4	98	245	3712
5	4-(CH_3_)	O	25	90	225	548
6	2-Cl-	O	9	95	237	1583
7	4-Br	O	4	98	245	3712
8	H	S	30	96	245	490
9	4-Cl	S	20	95	237	719
10	4-NO_3_	S	38	90	225	357
11	2,4-Cl_2_	S	23	95	237	625
12	4-(CH_3_)	S	40	91	227	344
13	2-Cl	S	35	94	235	405
14	4-Br	S	17	93	232	830

A mixture of aldehyde derivatives (1 mol), urea/thiourea (1.8 mol), malononitrile (1.2 mol) and bone char-nPrN-SO3H (0.4 mol%) were placed in a round-bottom flask in solvent-free conditions at 80 °C.

###  Recyclability of the catalyst

**Fig. 13:**
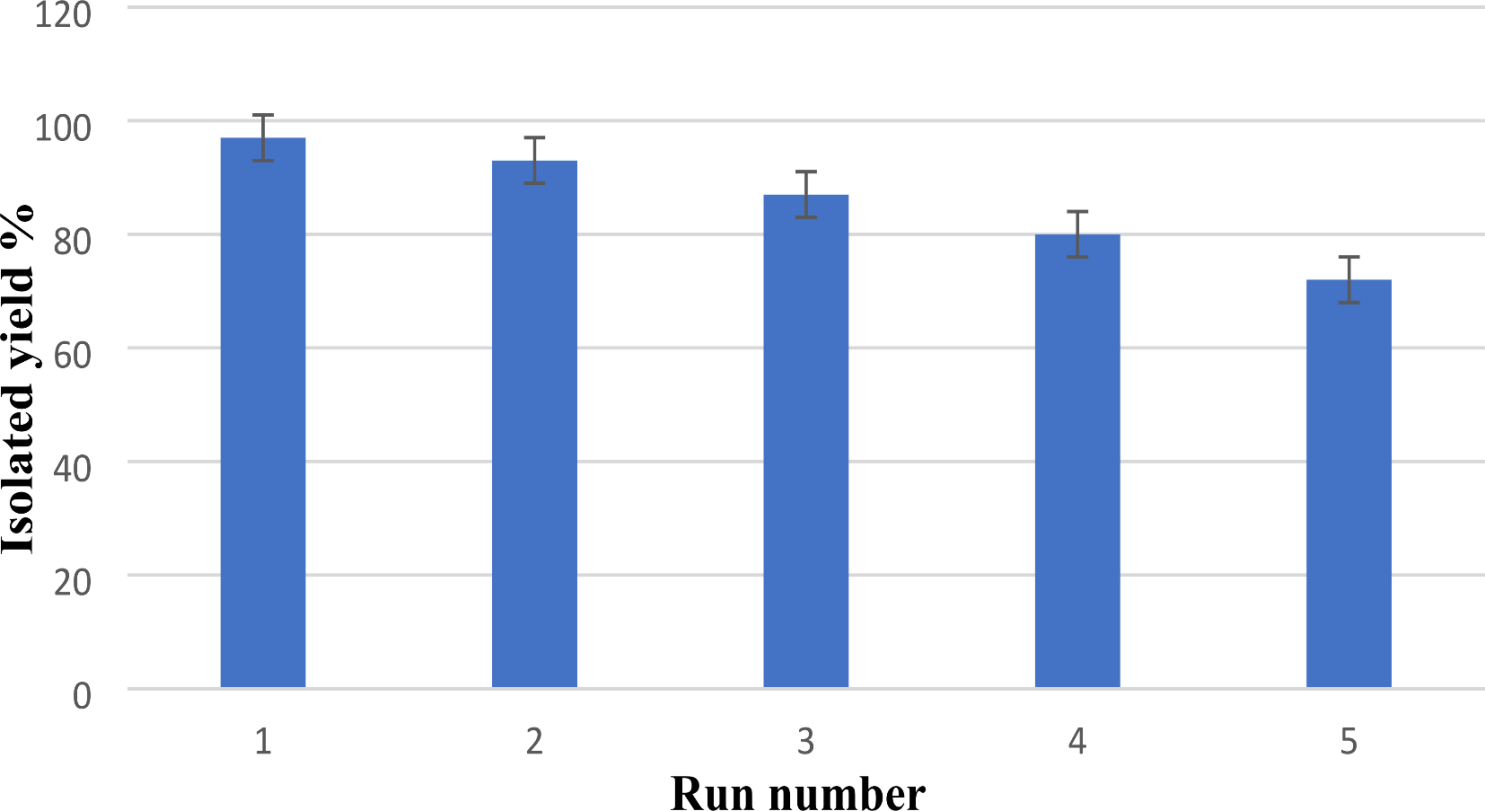
Recyclability of modified bone char as Bronsted acid catalyst in synthesis of pyrimidine-5-carbonitrile.

The recovering and recycling of the modified bone char were studied in the Synthesis of pyrimidine-5-carbonitrile using 4-chlorobenzaldehyde (1 mmol), malononitrile (1.2 mmol) and urea (1.8 mmol) in the presence bone char-nPrN-SO_3_H catalysis as model reaction. The resulting precipitate was filtered off and washed with hot ethanol (3 × 2 mL). The catalyst is recovered by simple filtration and washed several times with ethanol and water. The dried recovered catalyst was reused in the next experiment. The obtained results from recycling of modified bone char are summarized in Fig. 13. As shown this catalyst can be recycled up to 5 times without significantly loss in its catalytic activity. Also, Error bars indicate the range of data based on repeat experiments.

###  Comparison results of modified bone char with other catalysts for synthesis of 6-amino-5-cyano-4-(4chloro)-phenyl-2-mercapto pyrimidine

To extend the scope of merit of synthesized catalyst, it was compared with other catalysts for synthesis of 6-Amino-5-Cyano-4-(4Chloro)-Phenyl-2-Mercapto Pyrimidine (Table 4). The bone char catalyst shows very good reaction times and high yields. However, all reported methods have several disadvantages, including long reaction times, hard reaction conditions, and unfavorable results. Therefore, in this study, we used the cheapest and readily available natural based catalyst with the simple reaction method to synthesize pyrimidine-5-carbonitril.

**Table 4:** Comparison results of modified bone char with other catalysts for synthesis of 6-Amino-5-Cyano-4-(4Chloro)-Phenyl-2-Mercapto pyrimidine.

Entry	Condition	Time (h)	Yield (%)	Ref.
1	NH_4_Cl (ammonium chloride) (0.12 g), 110 °C, solvent free	4	81	44
2	Con. hydrochloric acid (0.3 ml), EtOH, Reflux	4	51	45
3	Sodium acetate (0.082 g), water, reflux	3.5	76	46
4	Phosphorus Pentoxide (0.05 g), EtOH, Reflux	1	92	47
5	CdFe_2_(C_4_H_4_O_6_)_3_.5H_2_O (0.02 g), EtOH, Reflux	45 min	92	48
6	Modified bone char (sulfonated bonchar) (0.05 g), 80 °C, solvent free	20 min	95	This work

## Conclusion

In this work, bone char was prepared from pyrolysis of bone cattle at 450 °C and applied as a catalyst support. Then functionalization by 3-aminopropyl-trietoxsysilan and sulfonation with chlorosulfonic acid made it an efficient and reusable heterogeneous solid acid biocatalyst for the one pot synthesis of pyrimidine-5-carbonitrile derivatives. Therefore, the first use of bone char as a catalyst support in multi compound organic reaction is the main novelty of this research. The presented biocatalyst was characterized by some techniques such as FT-IR, XRD, SEM, EDS, TGA, TEM and BET techniques.

## Electronic supplementary material

Below is the link to the electronic supplementary material.

Supplementary Material 1

## Data Availability

The datasets used and/or analyzed during the present study are available from the corresponding author upon reasonable request. All data generated or analyzed during this study are included in this published article [and its supplementary information file].

## Abbreviations

BC: Bone char
SEM: Scanning electron microscopy
TEM: Transmission Electron Microscopy
EDS: Energy-Dispersive X-ray spectroscopy
XRD: X-ray diffraction
TGA: Thermogravimetric analysis
FT-IR: Fourier transform infrared spectroscopy
