# Supplementary material for: Highly efficient synthesis of pyrimidine-5-carbonitrile derivatives over a robust biowaste bone char-Bronsted solid acid catalyst
Source: Sci Rep. 2024 Dec 6;14:30411. doi: 10.1038/s41598-024-82040-3 (PMC11621710; doi:10.1038/s41598-024-82040-3)
Supplement: Supplementary file 1 — Supplementary Material 1 [file 41598_2024_82040_MOESM1_ESM.docx]

**Highly efficient synthesis of pyrimidine-5-carbonitrile derivatives over a robust biowaste bone char-Bronsted solid acid catalyst**

**Zahra Siahpour and Maryam Hajjami ***

*Department of Organic Chemistry, Faculty of Chemistry and Petroleum Sciences, Bu-Ali Sina University, Hamedan 6517838683, Tel: +988138282807, Fax: +988138380709 Iran. m.hajjami@basu.ac.ir and mhajjami@yahoo.com (M. Hajjami)*

**6-Amino-4-(****4-boromophenyl)-5-cyano-2-hydroxypyrimidine**

**^1^H NMR (250 MHz, DMSO-*d*_6_) δ** **8.62 (s, 2H), 8.03 (d, *J* = 8.5 Hz, 2H), 7.90 (s, 1H), 7.70 (d, *J* = 8.6 Hz, 2H).**


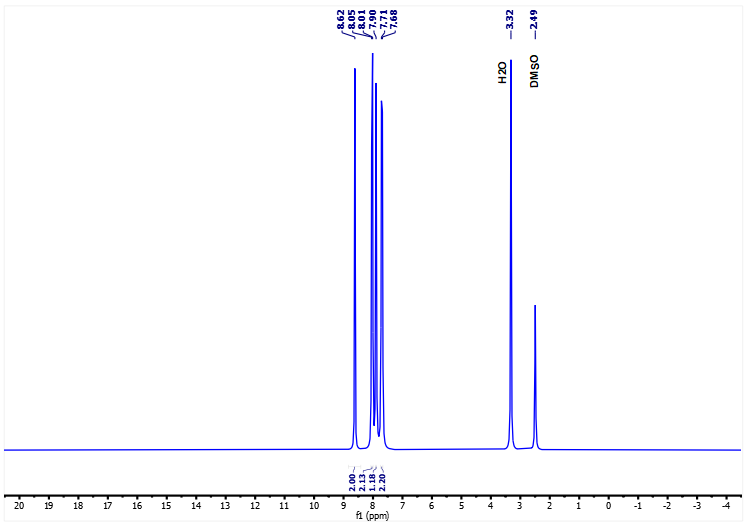

**Supplementary figure 1**

**6-Amino-4-(4-boromophenyl)-5-cyano-2-hydroxypyrimidine**

**^13^C NMR (63 MHz, DMSO) δ 157.20, 139.19, 135.83, 131.43, 130.50, 128.79, 113.77, 112.68, 87.55.**


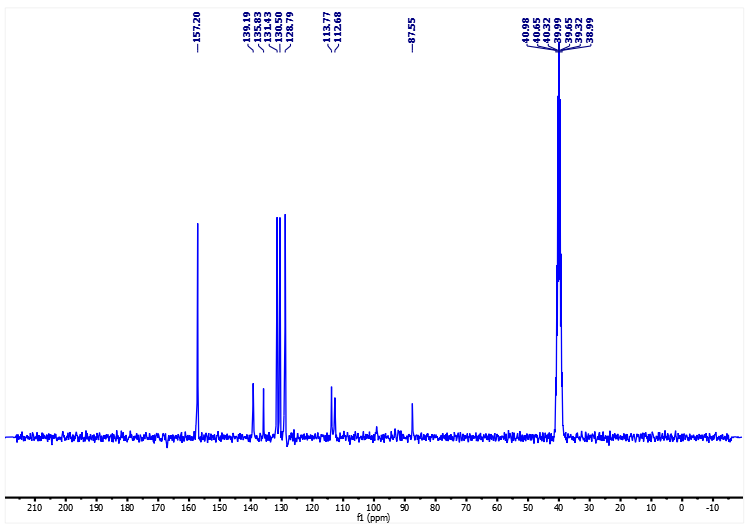

**Supplementary figure 2**

**6-Amino-4-(4-boromophenyl)-5-cyano-2-hydroxypyrimidine:**

**IR (KBr): υmax (cm^-1^) max 3400 (broad, OH), 3090 (NH_2_), 2227 and 1639 (CN).**


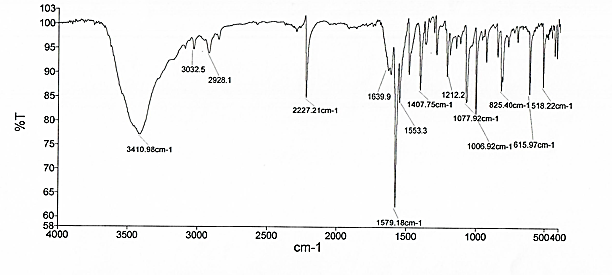

**Supplementary figure 3**

**6-Amino-4-(2,4-dichlorophenyl)-5-cyano-2-hydroxypyrimidine**

**^1^H NMR (250 MHz, DMSO-*d*_6_) δ 8.51 (s, 2H), 8.19 – 7.54 (m, 4H).**


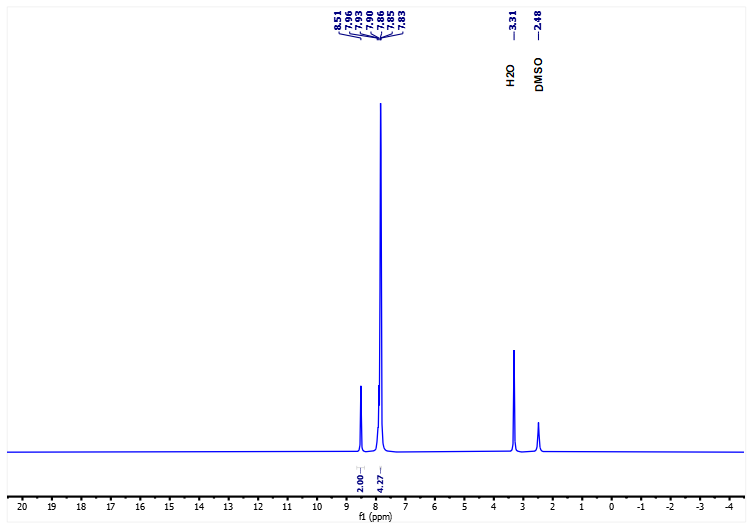

**Supplementary figure 4**

**6-Amino-4-(2,4-dichlorophenyl)-5-cyano-2-hydroxypyrimidine**

**^13^C NMR (63 MHz, DMSO) δ 182.26, 160.74, 134.81, 134.43, 133.14, 132.58, 130.84, 128.77, 114.52, 113.47, 82.78.**


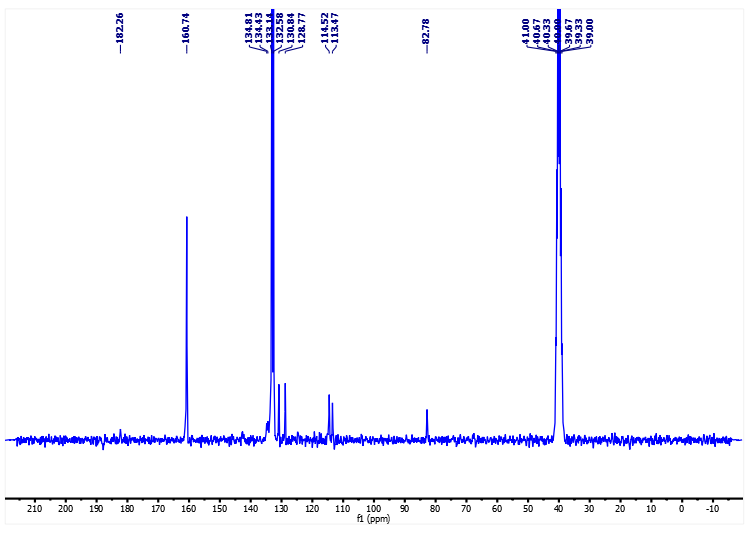

**Supplementary figure 5**

**6-Amino-4-(2,4-dichlorophenyl)-5-cyano-2-hydroxypyrimidine**:

**IR (KBr): υ_max_ (cm^-1^) max 3422 (broad, OH), 3102 (NH_2_), 2229 and 1639 (CN).**


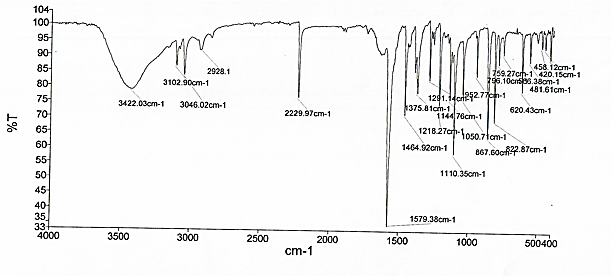

**Supplementary figure 6**

**6-Amino-5-Cyano-4-(4-boromo)-Phenyl-2-MercaptoPyrimidine**

**^1^H NMR (250 MHz, DMSO-*d*_6_) δ 8.50 (s, 2H), 7.86 (d, *J* = 7.4 Hz, 4H), 2.71 (s, 1H).**


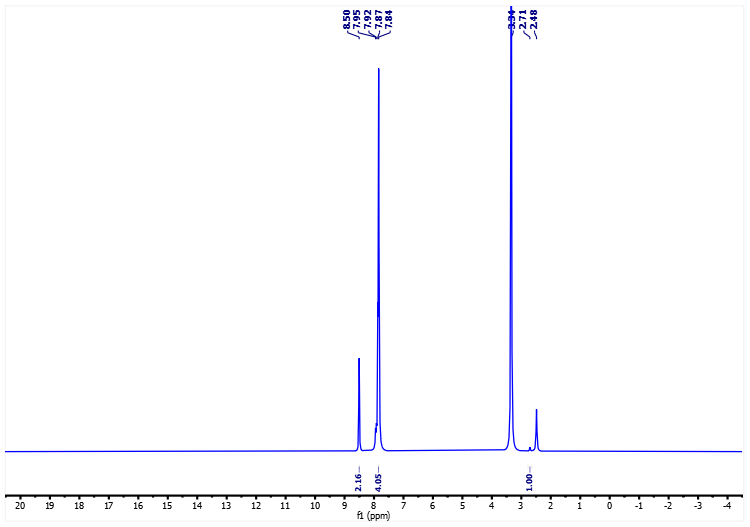

**Supplementary figure 7**

**6-Amino-5-Cyano-4-(4-boromo)-Phenyl-2-MercaptoPyrimidine**

**^13^C NMR (63 MHz, DMSO) δ 178.50, 161.97, 160.74, 133.12, 132.57, 130.84, 128.77, 114.52.**


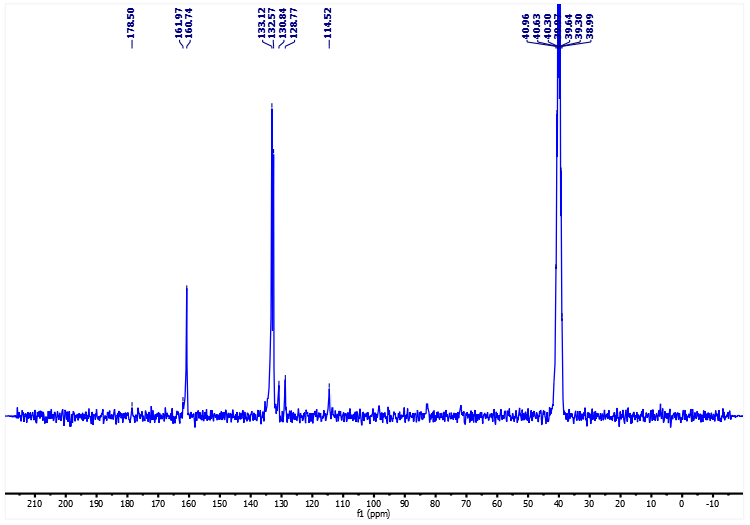

**Supplementary figure 8**

**6-Amino-5-Cyano-4-(4-boromo)-Phenyl-2-MercaptoPyrimidine:**

**IR (KBr): υ_max_ (cm^-1^) max, 3410 (NH_2_), 3032 (C-H), 2227 and 1639 (CN).**


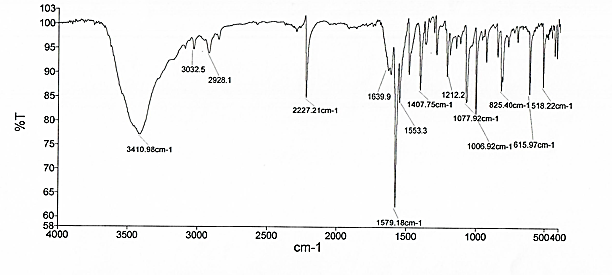

**Supplementary figure 9**
